# What Is Called a Blind Abscess?

**Published:** 1897-08

**Authors:** S. Alden Mills

**Affiliations:** New York


### WHAT IS CALLED A BLIND ABSCESS?

BY DR. S. ALDEN MILLS, NEW YORK.

WHAT can we demonstrate? This question was given some thought not long since in this JOURNAL, and in reading the statement of Dr. Louis Jack in the June number of the INTERNATIONAL DENTAL JOURNAL, page 372, that "when there is a fistula and an open foramen, or where we can *safely* make a small enlargement of the foramen, the case is reduced to a simplicity," etc.

Many know how much Dr. Atkinson was severely scored for teaching the opening of the foramen.

In this connection it may be well to refer to an uncalled-for slur, made at Saratoga last year, which has gone into the proceedings of the American Dental Association. It was, doubtless, aimed to antagonize the views held by the late Dr. Atkinson. He did teach boldly that the foramen should be opened, and that the disturbed territory should be broken up and reduced to a simple wound. This teaching can now be demonstrated as successful practice in a large proportion of cases, and if all could be under control, the success would practically be universal. Passing through the foramen in this manner is not a difficult operation, if performed with the intelligence all should have at command.

To enter into an elaborate technical description of this operation is deemed unnecessary. Apply an obtundent to the part through which it is proposed to make an artificial fistula, and then puncture with any suitable instrument. It is folly to deny that good results are not obtained by this operation. This has been demonstrated times without number in the writer's experience.

